# Protective efficacy of Ad26.COV2.S against SARS-CoV-2 B.1.351 in macaques

**DOI:** 10.1038/s41586-021-03732-8

**Published:** 2021-06-23

**Authors:** Jingyou Yu, Lisa H. Tostanoski, Noe B. Mercado, Katherine McMahan, Jinyan Liu, Catherine Jacob-Dolan, Abishek Chandrashekar, Caroline Atyeo, David R. Martinez, Tochi Anioke, Esther A. Bondzie, Aiquan Chang, Sarah Gardner, Victoria M. Giffin, David L. Hope, Felix Nampanya, Joseph Nkolola, Shivani Patel, Owen Sanborn, Daniel Sellers, Huahua Wan, Tammy Hayes, Katherine Bauer, Laurent Pessaint, Daniel Valentin, Zack Flinchbaugh, Renita Brown, Anthony Cook, Deandre Bueno-Wilkerson, Elyse Teow, Hanne Andersen, Mark G. Lewis, Amanda J. Martinot, Ralph S. Baric, Galit Alter, Frank Wegmann, Roland Zahn, Hanneke Schuitemaker, Dan H. Barouch

**Affiliations:** 1 Center for Virology and Vaccine Research, Beth Israel Deaconess Medical Center, Harvard Medical School, Boston, MA USA; 2 Harvard Medical School, Boston, MA USA; 3 Ragon Institute of MGH, MIT and Harvard, Cambridge, MA USA; 4 University of North Carolina at Chapel Hill, Chapel Hill, NC USA; 5 Tufts University Cummings School of Veterinary Medicine, North Grafton, MA USA; 6 Bioqual, Rockville, MD USA; 7 Janssen Vaccines & Prevention, Leiden, The Netherlands

**Keywords:** Vaccines, SARS-CoV-2

## Abstract

The emergence of SARS-CoV-2 variants that partially evade neutralizing antibodies poses a threat to the efficacy of current COVID-19 vaccines^1,2^. The Ad26.COV2.S vaccine expresses a stabilized spike protein from the WA1/2020 strain of SARS-CoV-2, and has recently demonstrated protective efficacy against symptomatic COVID-19 in humans in several geographical regions—including in South Africa, where 95% of sequenced viruses in cases of COVID-19 were the B.1.351 variant^3^. Here we show that Ad26.COV2.S elicits humoral and cellular immune responses that cross-react with the B.1.351 variant and protects against B.1.351 challenge in rhesus macaques. Ad26.COV2.S induced lower binding and neutralizing antibodies against B.1.351 as compared to WA1/2020, but elicited comparable CD8 and CD4 T cell responses against the WA1/2020, B.1.351, B.1.1.7, P.1 and CAL.20C variants. B.1.351 infection of control rhesus macaques resulted in higher levels of virus replication in bronchoalveolar lavage and nasal swabs than did WA1/2020 infection. Ad26.COV2.S provided robust protection against both WA1/2020 and B.1.351, although we observed higher levels of virus in vaccinated macaques after B.1.351 challenge. These data demonstrate that Ad26.COV2.S provided robust protection against B.1.351 challenge in rhesus macaques. Our findings have important implications for vaccine control of SARS-CoV-2 variants of concern.

## Abstract (web summary)

SARS-CoV-2 challenge of rhesus macaques demonstrates that the Ad26.COV2.S vaccine induces robust protection against both the WA1/2020 isolate and the B.1.351 variant of concern.

## Main

SARS-CoV-2 variants of concern have shown increased transmissibility and pathogenicity in humans^4,5^, and some variants have also demonstrated partial evasion of natural and vaccine-elicited neutralizing antibodies^1,2,6,7^. Ad26.COV2.S is a replication-incompetent human adenovirus type 26 vector^8^ that expresses a prefusion stabilized SARS-CoV-2 spike protein (S)^9,10^ from the Wuhan 2019 strain of SARS-CoV-2. It was previously reported that Ad26.COV2.S demonstrated protective efficacy against SARS-CoV-2 WA1/2020 challenges in hamsters and nonhuman primates^11–13^, and also showed safety and immunogenicity in humans^14,15^. A recent phase III efficacy trial has shown that Ad26.COV2.S provided 86%, 88% and 82% protection against severe COVID-19 disease by day 28 after vaccination in the USA, Brazil and South Africa, respectively^3^.

We developed a B.1.351 challenge stock by expansion of a seed stock (BEI Resources, NR-54974) in Calu-3 cells (ATCC HTB-55). We immunized 24 rhesus macaques in 4 experimental groups (*n* = 6 macaques per group) as follows: groups 1 and 3 received a sham vaccine (sham control macaques), and groups 2 and 4 received a single immunization with 5 × 10^10^ viral particles of Ad26.COV2.S; after vaccination, groups 1 and 2 were challenged with the original SARS-CoV 2 strain WA1/2020, and groups 3 and 4 were challenged with the SARS-CoV-2 variant B.1.351.

## Ad26.COV2.S immunogenicity and cross-reactivity

**Fig. 1:**
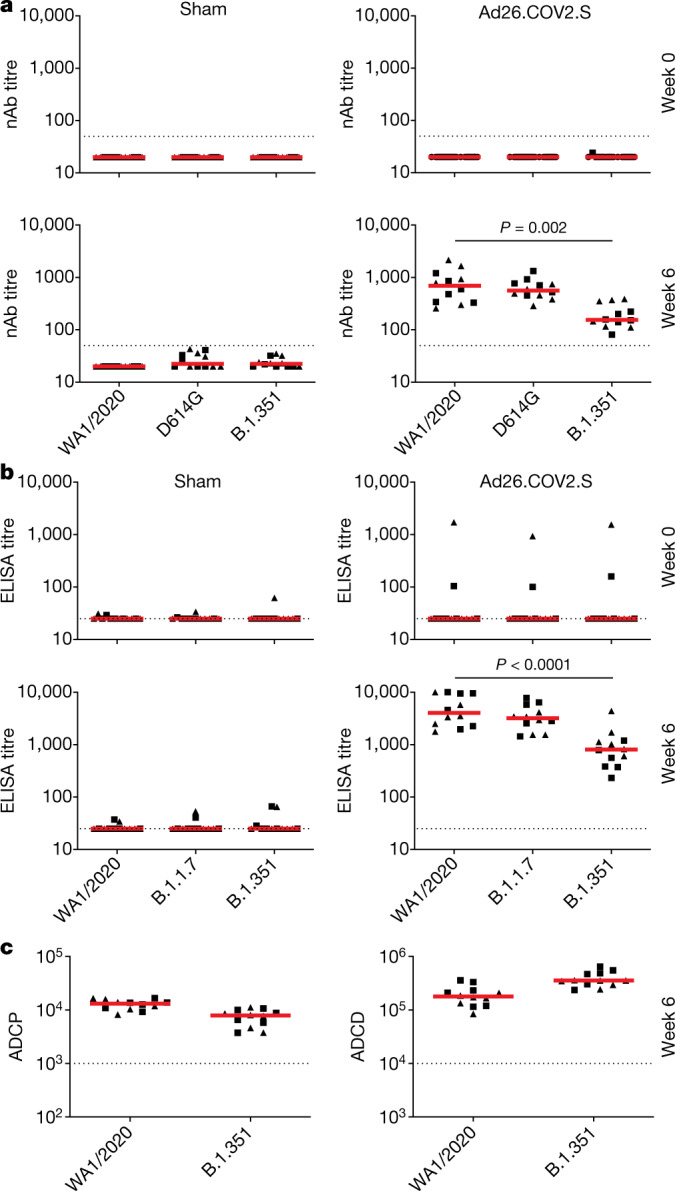
Antibody responses in vaccinated rhesus macaques. **a**, Pseudovirus neutralizing antibody (nAb) assays against the SARS-CoV-2 WA1/2020, D614G and B.1.351 variants were assessed at week 0 (top panels) and week 6 (bottom panels) in macaques that received a single immunization of sham vaccine (left panels) or 5 × 10^10^ viral particles of Ad26.COV2.S (right panels). **b**, RBD-specific binding antibody responses of sham control (left panels) or Ad26.COV2.S-vaccinated (right panels) macaques against WA1/2020, B.1.1.7, and B.1.351 were assessed by ELISA at week 0 (top panels) and week 6 (bottom panels). **c**, Antibody-dependent cellular phagocytosis (ADCP) (phagocytic score) and antibody-dependent complement deposition (ADCD) (mean fluorescence intensity) were evaluated against WA1/2020 and B.1.351 at week 6. Macaques that eventually were challenged with WA1/2020 (triangles) or B.1.351 (squares) are depicted. Horizontal red bars reflect median responses. *P* values reflect two-sided Wilcoxon rank-sum tests. Dotted lines reflect the limits of quantification of the assay. *n* = 24 independent samples (12 sham and 12 Ad26.COV2.S). Source data

**Extended Data Fig. 1:**
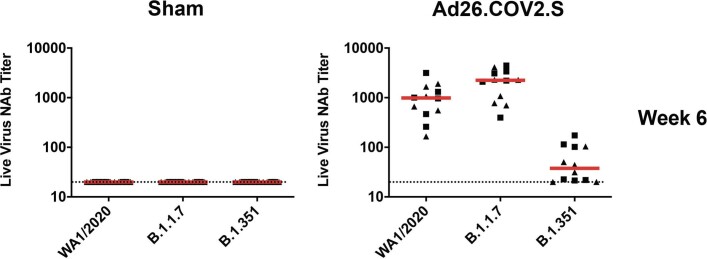
Live virus neutralizing antibody responses in vaccinated rhesus macaques. Live virus neutralizing antibody responses against the SARS-CoV-2 WA1/2020, B.1.1.7 and B.1.351 variants were assessed at week 6 in macaques that received a single immunization of sham vaccine or 5 × 10^10^ viral particles of Ad26.COV2.S. Macaques that eventually were challenged with WA1/2020 (triangles) or B.1.351 (squares) are depicted. Horizontal red bars reflect median responses. Dotted lines reflect assay limits of quantification. *n* = 24 independent samples (12 sham, 12 Ad26.COV2.S).

We assessed vaccine-induced antibody responses against the SARS-CoV-2 WA1/2020 strain as well as against B.1.351. Using a luciferase-based pseudovirus neutralizing antibody assay^12,16–18^, we found that the median neutralizing antibody titres in macaques that received Ad26.COV2.S vaccine were less than 20 at week 0, and were 693, 561, and 155 against the WA1/2020, D614G and B.1.351 strains, respectively, in Ad26.COV2.S-vaccinated macaques at week 6 (Fig. 1a). These data show a median 4.5-fold reduction of neutralizing antibody titres against B.1.351 as compared to WA1/2020 (*P* = 0.0002, Wilcoxon rank-sum test). Live-virus neutralizing antibody assays^19^ showed a greater reduction of neutralizing antibody titres against B.1.351 (Extended Data Fig. 1).

**Extended Data Fig. 2:**
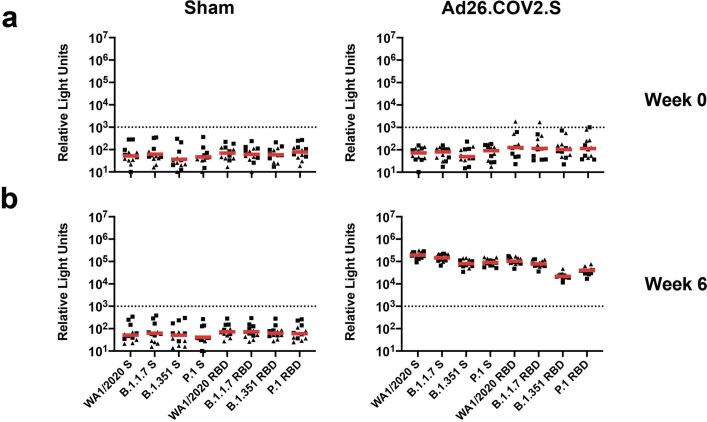
Binding antibody responses in vaccinated rhesus macaques by ECLA. **a**, **b**, S- and RBD-specific binding antibody responses against the SARS-CoV-2 WA1/2020, B.1.1.7, B.1.351 and P.1 variants were assessed by ECLA at week 0 (**a**) and week 6 (**b**) in macaques that received a single immunization of sham-negative control or 5 × 10^10^ viral particles of Ad26.COV2.S. Macaques that eventually were challenged with WA1/2020 (triangles) or B.1.351 (squares) are depicted. Horizontal red bars reflect median responses. Dotted lines reflect assay limits of quantification.

Median receptor-binding domain (RBD)-specific enzyme-linked immunosorbent assay (ELISA) titres in macaques that received Ad26.COV2.S vaccine were less than 25 at week 0, and were 4,050, 3,186 and 805 against the WA1/2020, B.1.1.7 and B.1.351 strains, respectively, in Ad26.COV2.S-vaccinated macaques at week 6 (Fig. 1b). These data show a median 5.0-fold reduction of RBD-specific ELISA titres against B.1.351 as compared to WA1/2020 (*P* < 0.0001, Wilcoxon rank-sum test). We also used an electrochemiluminescence assay (ECLA)^20^ to evaluate S- and RBD-specific binding antibody responses to WA1/2020, B.1.1.7, P.1. and B.1.351 (Extended Data Fig. 2). Similar to the ELISA titres, median RBD-specific ECLA responses were reduced against P.1 and B.1.351 as compared to WA1/2020 at week 6, whereas we observed a smaller effect with S-specific ECLA responses. Antibody-dependent cellular phagocytosis and antibody-dependent complement deposition responses^21^ were more comparable against WA1/2020 and B.1.351 than were ELISA titres or ECLA responses (Fig. 1c).

**Fig. 2:**
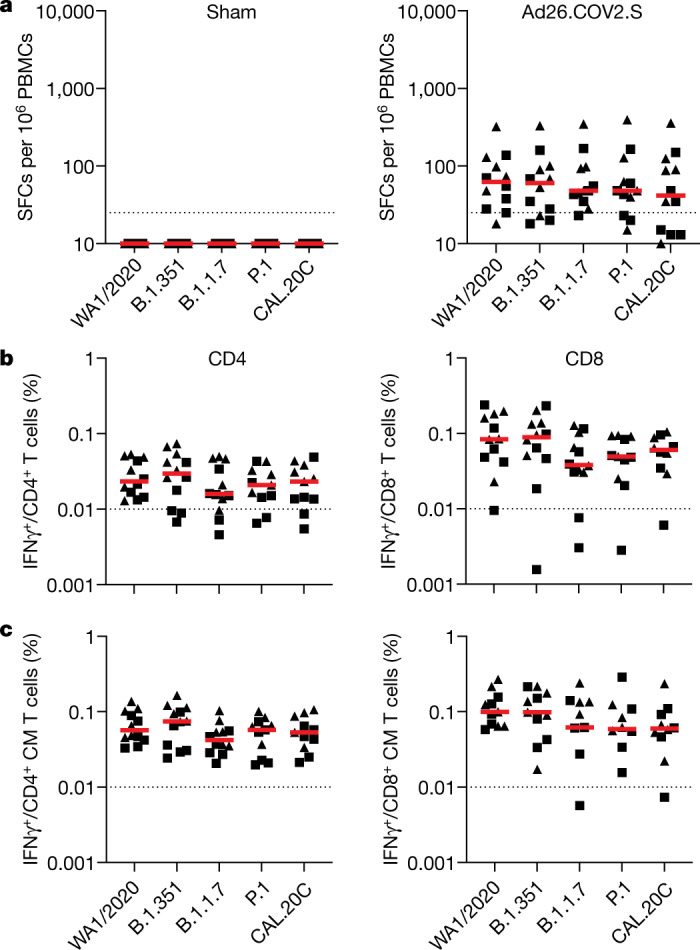
T cell responses in vaccinated rhesus macaques. **a**–**c**, Cellular immune responses to pooled S peptides of sham control (left) or Ad26.COV2.S-vaccinated (right) macaques were assessed by IFNγ ELISPOT assays at week 4 (**a**) and IFNγ intracellular cytokine staining assays at week 6 (**b**, **c**) to WA1/2020, B.1.351, B.1.1.7, P.1, and CAL.20C variants. Intracellular cytokine staining assays show IFNγ responses in CD4^+^ (left) and CD8^+^ (right) T cells (**b**) and CD28^+^CD95^+^CD4^+^ (left) or CD28^+^CD95^+^CD8^+^ (right) central memory (CM) T cells (**c**). Macaques that eventually were challenged with WA1/2020 (triangles) or B.1.351 (squares) are depicted. Horizontal red bars reflect median responses. Dotted lines reflect assay limits of quantification. *n* = 24 independent samples (12 sham and 12 Ad26.COV2.S). SPCs, spot-forming cells; PBMCs, peripheral blood mononuclear cells. Source data

We assessed S-specific cellular immune responses using pooled peptide IFNγ enzyme-linked immunospot (ELISPOT) assays in peripheral blood mononuclear cells at week 4. ELISPOT responses were comparable among the WA1/2020, B.1.351, B.1.1.7, P.1 and CAL.20C strains, with no evidence of decreased responses against the variants (Fig. 2a). We also evaluated S-specific CD8^+^ and CD4^+^ T cell responses using multi-parameter intracellular cytokine staining assays at week 6 (Supplementary Fig. 1). IFNγ CD8^+^ and CD4^+^ T cell responses were comparable among the WA1/2020, B.1.351, B.1.1.7, P.1 and CAL.20C strains (Fig. 2b). Similarly, IFNγ central memory CD28^+^CD95^+^CD4^+^ and CD28^+^CD95^+^CD8^+^ T cell responses were comparable across these variants (Fig. 2c). These data show that S-specific cellular immune responses were comparable for these SARS-CoV-2 variants.

## Homologous and heterologous SARS-CoV-2 challenges

**Fig. 3:**
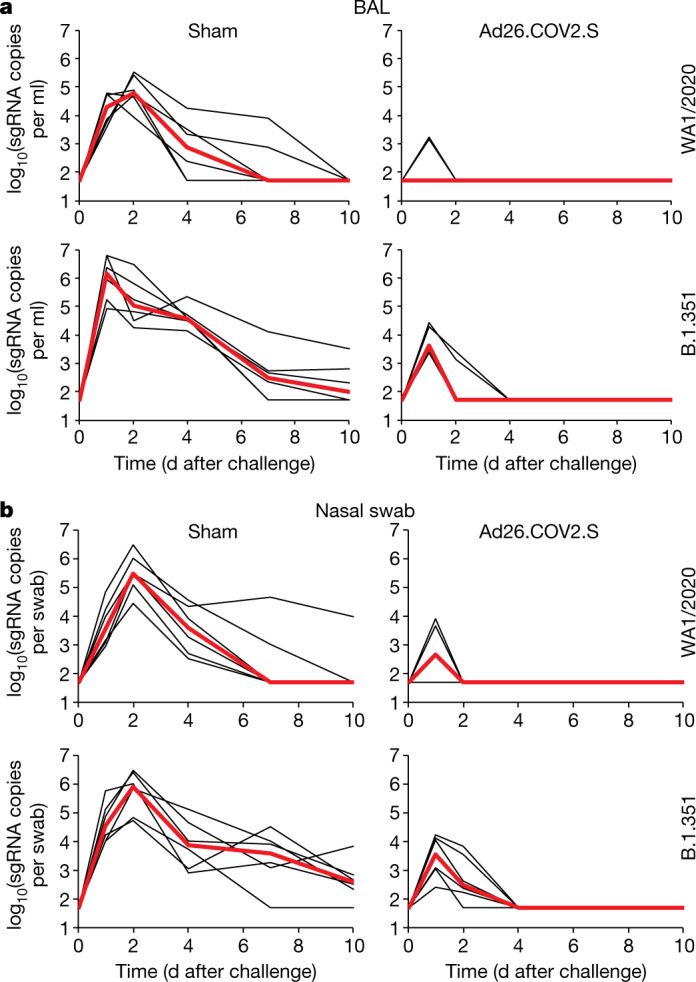
Protective efficacy after SARS-CoV-2 challenge. Rhesus macaques were challenged by the intranasal and intratracheal routes with 5 × 10^5^ TCID_50_ SARS-CoV-2 WA1/2020 or B.1.351. **a**, log_10_(sgRNA copies per ml) (limit of quantification of 50 copies per ml) are shown in BAL of sham control (left) or Ad26.COV2.S-vaccinated (right) macaques after challenge with WA1/2020 (top) or B.1.351 (bottom). **b**, log_10_(sgRNA copies per swab) (limit of quantification 50 copies per swab) are shown in nasal swabs of sham control (left) or Ad26.COV2.S-vaccinated (right) macaques after challenge with WA1/2020 (top) or B.1.351 (bottom). Red lines reflect median values. *n* = 24 independent samples (12 sham and 12 Ad26.COV2.S). Source data

We challenged all macaques at week 6 with a 5 × 10^5^ 50% tissue culture infectious dose (TCID_50_) of SARS-CoV-2 WA1/2020^12,16,17,22^ or B.1.351 by the intranasal and intratracheal routes. We assessed viral loads in bronchoalveolar lavage (BAL) and nasal swabs by reverse-transcription PCR (RT–PCR) specific for subgenomic mRNA (sgRNA), which is believed to measure replicating virus^16,23,24^. All sham control macaques were infected and showed higher median peak sgRNA of 6.16 (range of 4.93–6.80) log_10_(sgRNA copies per ml) in BAL for B.1.351, as compared to 4.80 (range of 4.70–5.52) log_10_(sgRNA copies per ml) for WA1/2020 (Fig. 3a). By contrast, vaccinated macaques demonstrated a median peak of 3.62 (range of 3.37–4.43) log_10_(sgRNA copies per ml) in BAL for B.1.351, as compared with less than 1.69 (range of <1.69 to 3.23) log_10_(sgRNA copies per ml) in BAL for WA1/2020 (Fig. 3a). Sham control macaques also showed a trend towards a higher median peak sgRNA of 5.90 (range of 4.73–6.47) log_10_(sgRNA copies per swab) in nasal swabs for B.1.351, as compared with 5.48 (range of 4.44–6.00) log_10_(sgRNA copies per swab) for WA1.2020 (Fig. 3b). Vaccinated macaques demonstrated a median peak of 3.57 (range of 2.41–4.21) log_10_(sgRNA copies per swab) in nasal swabs for B.1.351, as compared with 2.64 (range of <1.69 to 3.89) log_10_(sgRNA copies per swab) in nasal swabs for WA1/2020 (Fig. 3b).

**Fig. 4:**
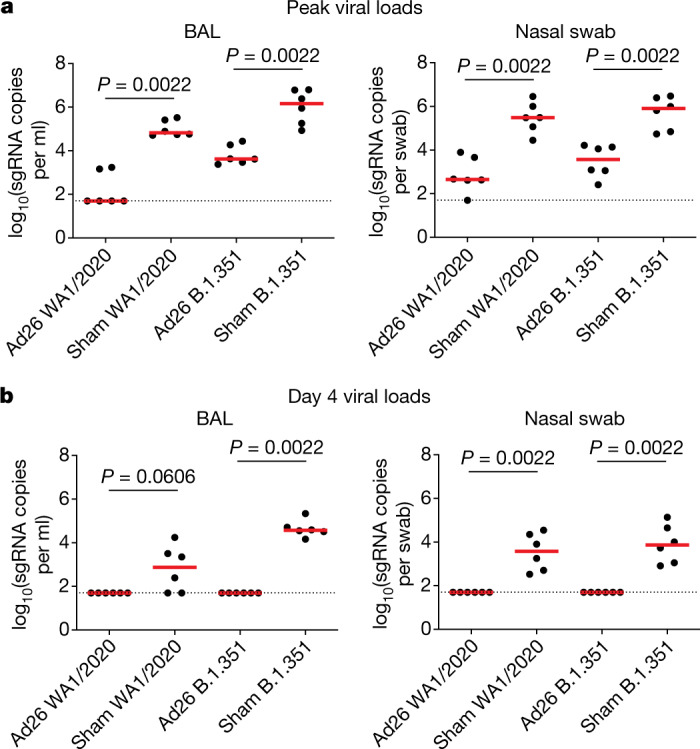
Summary of protective efficacy after SARS-CoV-2 challenge. **a**, **b**, Peak (**a**) and day 4 (**b**) viral loads in BAL (left) and nasal swabs (right) of sham control (sham) and Ad26.COV2.S-vaccinated (Ad26) macaques after challenge with WA1/2020 or B.1.351. Horizontal red bars reflect median values. *P* values reflect two-sided Wilcoxon rank-sum tests. Dotted lines reflect the limits of quantification of the assay. *n* = 24 independent samples (12 sham and 12 Ad26.COV2.S). Source data

**Extended Data Fig. 3:**
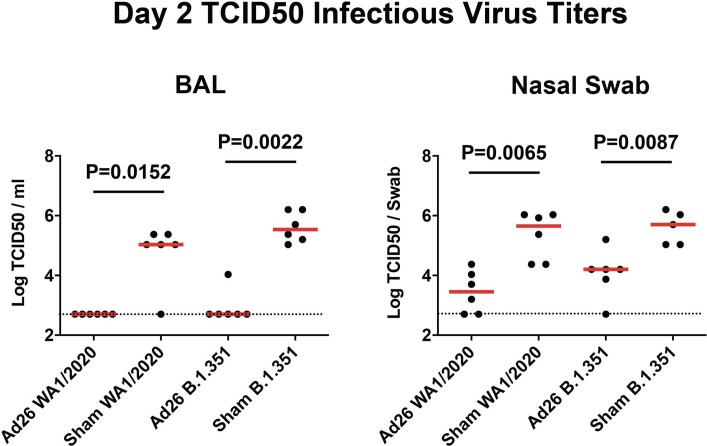
Infectious virus titres after SARS-CoV-2 challenge. Day 2 infectious virus titres by TCID_50_ assays in BAL and nasal swabs after challenge. Horizontal red bars reflect median values. *P* values reflect two-sided Wilcoxon rank-sum tests. Dotted lines reflect assay limits of quantification. *n* = 24 independent samples (12 sham, 12 Ad26.COV2.S).

B.1.351 led to higher peak viral loads, faster kinetics of viral replication and a longer duration of viral replication as compared with WA1/2020 in sham control macaques, which suggests that B.1.351 is a more stringent challenge in the macaque model. Ad26.COV2.S provided robust protection against peak viral replication for both strains, including a 3.13 and 2.54 log reduction of peak sgRNA copies per ml in BAL for WA1/2020 and B.1.351, respectively, and a 2.84 and 2.33 log reduction of peak sgRNA copies per swab in nasal swabs for WA1/2020 and B.1.351, respectively (*P* = 0.0022 for both BAL and nasal swabs for both WA1/2020 and B.1.351, Wilcoxon rank-sum tests) (Fig. 4a). By day 4 after challenge, viral loads were undetectable in Ad26.COV2.S-vaccinated macaques after both WA1/2020 and B.1.351 challenge, whereas viral loads were positive in most sham control macaques for WA1/2020 and in all sham control macaques for B.1.351 (Fig. 4b). Ad26.COV2.S also provided similar robust protection against day 2 infectious virus titres, as assessed by TCID_50_ assays (Extended Data Fig. 3).

## Correlates of protection

**Extended Data Fig. 4:**
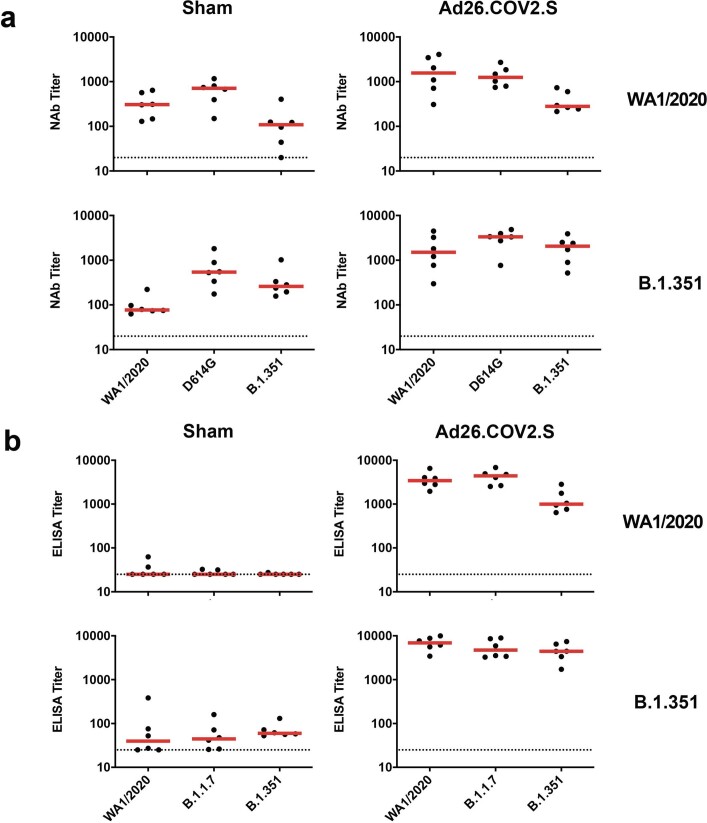
Binding and neutralizing antibody responses in challenged rhesus macaques. **a**, **b**, Pseudovirus neutralizing antibody assays against the SARS-CoV-2 WA1/2020, D614G, and B.1.351 variants were assessed (**a**) and RBD-specific binding antibody responses against the SARS-CoV-2 WA1/2020, B.1.1.7, and B.1.351 variants were assessed by ELISA (**b**) on day 10 after challenge in macaques that received a single immunization of sham vaccine or 5 × 10^10^ viral particles of Ad26.COV2.S. Macaques that were challenged with WA1/2020 or B.1.351 are shown in separate graphs. Horizontal red bars reflect median responses. Dotted lines reflect assay limits of quantification. *n* = 24 independent samples (12 sham, 12 Ad26.COV2.S).

**Extended Data Fig. 5:**
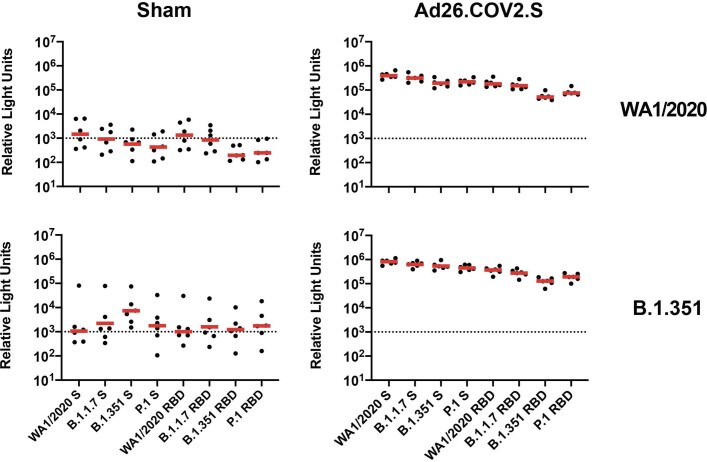
Binding antibody responses in challenged rhesus macaques by ECLA. S- and RBD-specific binding antibody responses against the SARS-CoV-2 WA1/2020, B.1.1.7, B.1.351 and P.1 variants were assessed by ECLA on day 10 after challenge in macaques that received a single immunization of sham vaccine or 5 × 10^10^ viral particles of Ad26.COV2.S. Macaques that were challenged with WA1/2020 or B.1.351 are shown in separate graphs. Horizontal red bars reflect median responses. Dotted lines reflect assay limit of quantification. *n* = 24 independent samples (12 sham, 12 Ad26.COV2.S).

**Extended Data Fig. 6:**
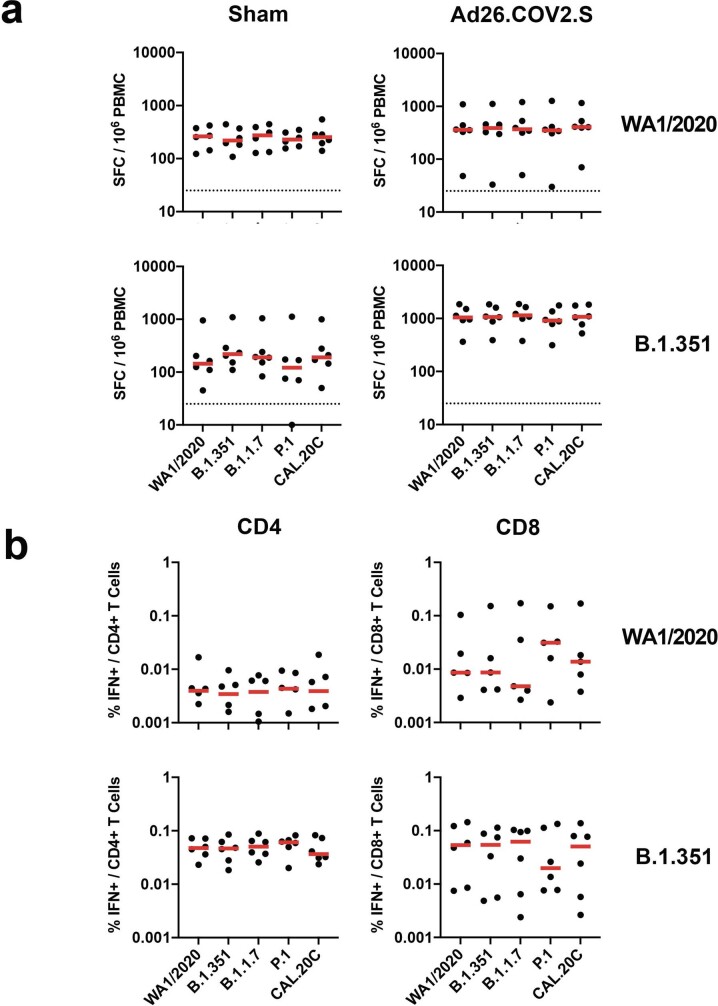
T cell responses in vaccinated rhesus macaques by ELISPOT assays. **a**, Cellular immune responses to pooled S peptides were assessed by IFNγ ELISPOT assays on day 10 after challenge to WA1/2020, B.1.351, B.1.1.7, P.1 and CAL.20C variants. **b**, CD4^+^ and CD8^+^ T cell responses to pooled S peptides were assessed by IFNγ intracellular cytokine staining assays on day 10 after challenge to WA1/2020, B.1.351, B.1.1.7, P.1 and CAL.20C variants. Horizontal red bars reflect median responses. *n* = 24 independent samples (12 sham, 12 Ad26.COV2.S).

On day 10 after challenge (study week 8), sham control macaques developed both humoral and cellular immune responses, as expected (Extended Data Figs. 4–6). In sham control macaques, WA1/2020 challenge led to higher neutralizing antibody titres to WA1/2020 than to B.1.351, whereas B.1.351 challenge led to higher neutralizing antibody titres to B.1.351 than to WA1/2020 (Extended Data Fig. 4a), and cellular responses were comparable across all strains regardless of the challenge virus (Extended Data Fig. 6), consistent with the vaccine immunogenicity data. Ad26.COV2.S-vaccinated macaques developed increased humoral and cellular immune responses after challenge. The low ELISA titres in sham control macaques probably reflect the early (day 10) time point after challenge (Extended Data Fig. 4b).

**Extended Data Fig. 7:**
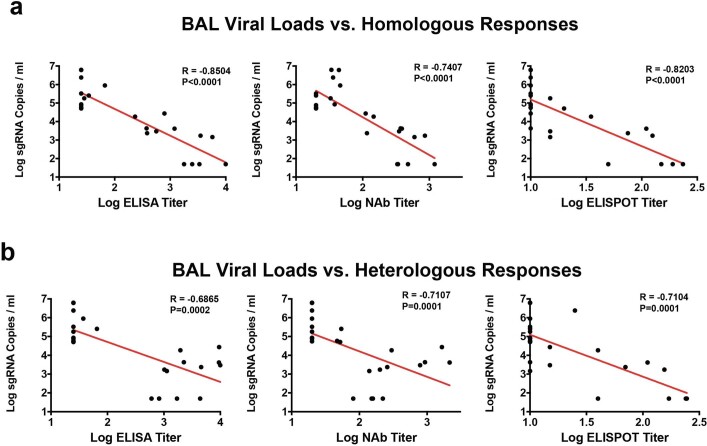
Correlates of protection in BAL. **a**, **b**, Correlations of log(peak sgRNA copies per ml) in BAL after challenge versus log-transformed ELISA titres, neutralizing antibody titres or ELISPOT responses to the homologous (**a**) or heterologous (**b**) challenge virus (WA1/2020, B.1.351) at week 6 after vaccination. Red lines reflect the best linear fit relationship between these variables. *P* and *R* values reflect two-sided Spearman rank-correlation tests.

**Extended Data Fig. 8:**
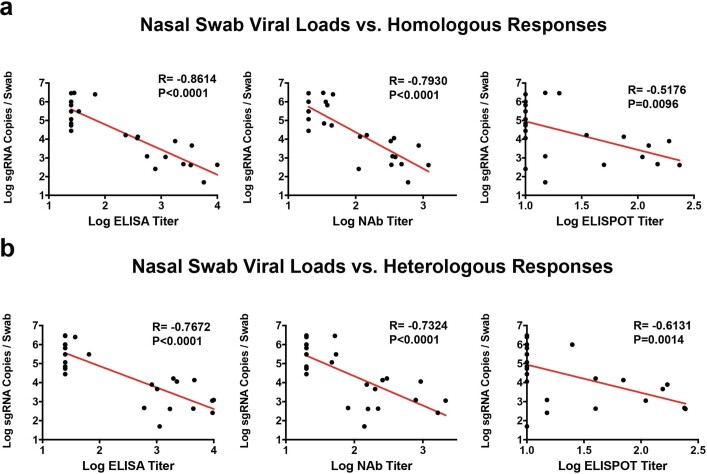
Correlates of protection in nasal swabs. **a**, **b**, Correlations of log(peak sgRNA copies per ml) in nasal swabs after challenge versus log-transformed ELISA titres, neutralizing antibody titres or ELISPOT responses to the homologous (**a**) or heterologous (**b**) challenge virus (WA1/2020, B.1.351) at week 6 after vaccination. Red lines reflect the best linear fit relationship between these variables. *P* and *R* values reflect two-sided Spearman rank-correlation tests.

Peak log_10_(sgRNA) in BAL (Extended Data Fig. 7) and in nasal swabs (Extended Data Fig. 8) after challenge inversely correlated with log_10_ ELISA, neutralizing antibody and ELISPOT responses at week 6, which suggests that both antibody and T cell responses correlate with protection. Correlations were slightly stronger for immune responses against the homologous challenge virus as compared with the heterologous challenge virus.

## Histopathology

**Fig. 5:**
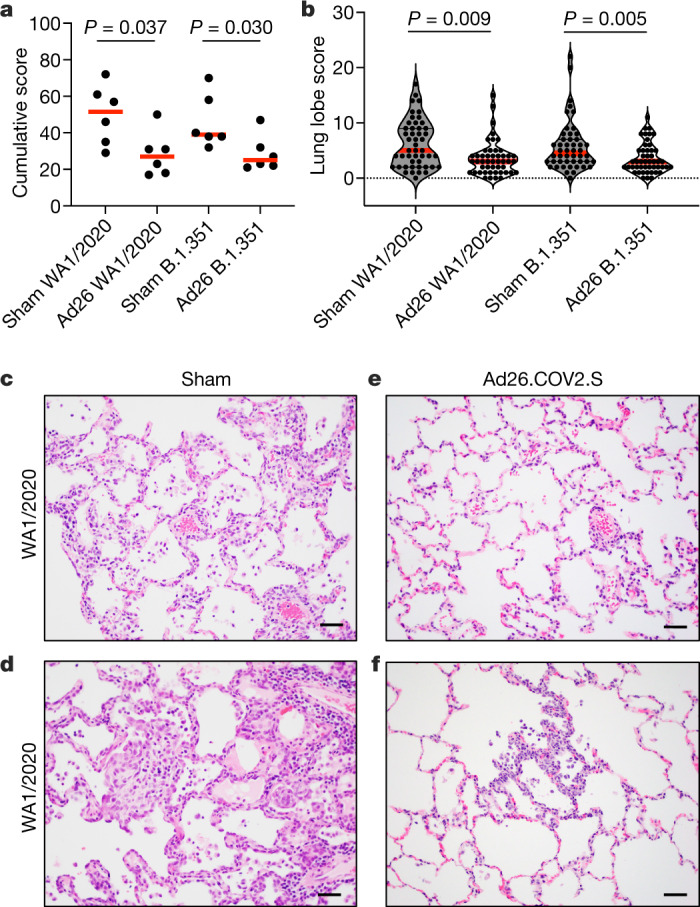
Histopathology after SARS-CoV-2 challenge. **a**, Cumulative histopathologic scoring of lung lesions from eight representative lung lobes from Ad26.COV2.S-vaccinated (Ad26) and sham control (sham) macaques on day 10 after challenge with WA1/2020 or B.1.351 SARS CoV-2 variants. **b**, Eight representative samples from cranial, middle and caudal lung lobes from the left and right lungs were evaluated from each macaque, and were scored independently for each of the following lesions: interstitial inflammation and septal thickening, interstitial infiltrate (eosinophils), interstitial infiltrate (neutrophils), hyaline membranes, interstitial fibrosis, alveolar infiltrate (macrophages), bronchoalveolar infiltrate (neutrophils), epithelial syncytia, type II pneumocyte hyperplasia, bronchi infiltrate (macrophages), bronchi infiltrate (neutrophils), bronchi (hyperplasia of bronchus-associated lymphoid tissue), bronchiolar or peribronchiolar infiltrate (mononuclear cells), perivascular infiltrate (mononuclear cells) and endothelialitis. Each feature assessed was assigned a score of: 0, no substantial findings; 1, minimal; 2, mild; 3, moderate; 4, moderate to severe; 5, marked or severe. Scores were added for all lesions across all lung lobes for each macaque, for a maximum possible score of 600 for each macaque. Horizontal red lines reflect median values. *P* values reflect two-sided Wilcoxon rank-sum tests. **c**–**f**, Representative lung histopathology from at least eight evaluated tissues from sham control (**c**, **d**) and Ad26.COV2.S-vaccinated (**e**, **f**) macaques challenged with WA1/2020 (**c**, **e**) or B.1.351 (**d**, **f**) (on day 10 after the challenge), showing increased alveolar macrophages and thickened alveolar septa with inflammatory infiltrates and fibrosis (**c**), increased alveolar macrophages and epithelial syncytia within alveolar spaces, thickened and fibrotic alveolar septa with inflammatory infiltrates, focal alveolar and perivascular inflammatory infiltrates (**d**), focal perivascular inflammation (**e**) and focal expansion of alveolar septa with inflammatory infiltrates (**f**). Lungs evaluated were inflated or suffused with 10% formalin. In **c**–**f**, tissues were stained with haematoxylin and eosin. Scale bars, 20 μm. *n* = 24 independent samples (12 sham and 12 Ad26.COV2.S) (**a**, **b**); *n* = 4 representative samples (2 sham and 2 Ad26.COV2.S) (**c**–**f**). Source data

**Extended Data Fig. 9:**
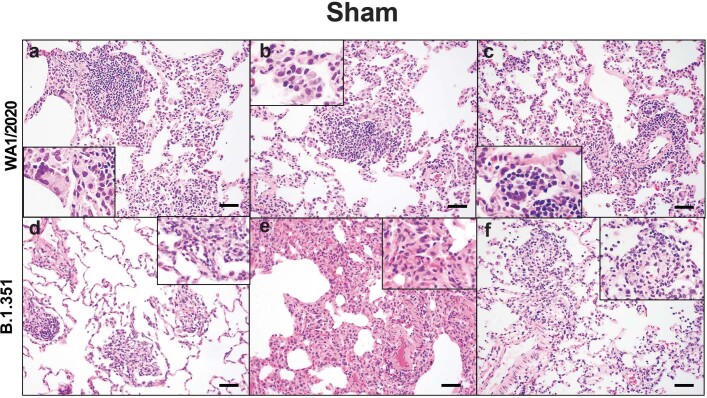
Representative histopathology in sham control macaques after SARS-CoV-2 challenge. **a**–**f**, Locally extensive moderate-to-severe lesions were observed in sham control macaques challenged with WA1/2020 (**a**–**c**) or B.1.351 (**d**–**f**), on day 10 after challenge. **a**, Syncytia, lymphoid proliferation and locally extensive interstitial inflammation. **b**, Type II pneumocyte hyperplasia and lymphoid proliferation. **c**, Perivascular alveolar infiltrates and interstitial inflammation. **d**, Alveolar macrophage infiltrates. **e**, Severe mononuclear alveolar infiltrates and pneumocyte hyperplasia. **f**, Perivascular infiltrates and interstitial inflammation. At least eight tissues were assessed per macaques. Haematoxylin and eosin staining. Scale bars, 20 μm.

**Extended Data Fig. 10:**
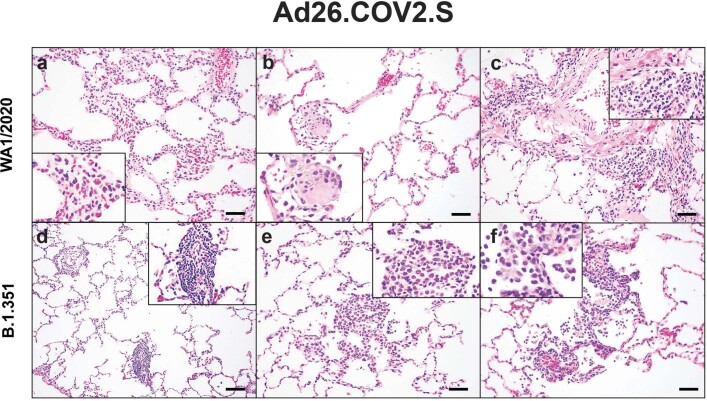
Representative histopathology in Ad26.COV2.S-vaccinated macaques after SARS-CoV-2 challenge. **a**–**f**, Focal minimal-to-mild lesions were observed in Ad26.COV2.S-vaccinated macaques challenged with WA1/2020 (**a**–**c**) or B.1.351 (**d**–**f**), on day 10 after challenge. **a**, Interstitial inflammation. **b**, Syncytia. **c**, Perivascular neutrophilic infiltrates. **d**, Perivascular mononuclear inflammation. **e**, Type II pneumocyte hyperplasia. **f**, Alveolar macrophage infiltrates. At least eight tissues were assessed per macaque. Haematoxylin and eosin staining. Scale bar, 20 μm.

Ad26.COV2.S-vaccinated macaques demonstrated reduced lung histopathology compared with sham control macaques at necropsy on day 10 after WA1/2020 or B.1.351 challenge (Fig. 5a, b), although viral replication had largely resolved by day 10. Sham control macaques infected with WA1/2020 and B.1.351 had histopathological lesions that were consistent with previous reports^16^, including focal to locally extensive interstitial pneumonia with neutrophilic and mononuclear interstitial infiltrates, alveolar syncytia, and increased numbers of alveolar macrophages. Perivascular inflammation and type II pneumocyte hyperplasia were prominent features in both groups of sham control macaques, as were multifocal regions of fibrosis (Fig. 5c, d, Extended Data Fig. 9). Ad26.COV2.S-vaccinated macaques had only rare lesions, predominantly small and focal regions of interstitial inflammation, and rare syncytia in isolated lung lobes (Fig. 5e, f, Extended Data Fig. 10). No evidence of eosinophilic infiltrates or enhanced respiratory disease was observed in Ad26.COV2.S-vaccinated macaques.

## Discussion

It has previously been reported that Ad26.COV2.S provided robust protection against challenge with SARS-CoV-2 WA1/2020 in both rhesus macaques and hamsters^11–13^. In this study, we show that Ad26.COV2.S induced cross-reactive antibody and T cell responses against SARS-CoV-2 variants of concern—including the B.1.351 variant, which has several mutations (including E484K) that lead to partial evasion of natural and vaccine-elicited neutralizing antibodies^1,2,6,7^. Binding and neutralizing antibody titres were suppressed 4–5-fold against B.1.351 as compared to WA1/2020, but Fc functional antibody responses were affected less, and T cell responses were not affected at all by the SARS-CoV-2 variants. Ad26.COV2.S provided robust protection against both high-dose WA1/2020 and B.1.351 challenges. These data have important implications for the potential utility of current vaccines and inform boosting strategies against SARS-CoV-2 variants of concern.

Our data are consistent with findings in humans in a recent phase III clinical trial of Ad26.COV2.S that was conducted in the USA, Latin America (Argentina, Brazil, Chile, Colombia, Mexico and Peru) and South Africa^3^. Robust protection was observed in all geographical regions, with similar levels of protection against severe COVID-19 disease regardless of variant, including in the USA, in Brazil (where 69% of cases with sequence data were the P.2 variant) and in South Africa (where 95% of cases with sequence data were the B.1.351 variant). In the current study in macaques, B.1.351 infection led to a higher magnitude of and more prolonged viral replication in the upper and lower respiratory tracts than did WA1/2020. Nevertheless, Ad26.COV2.S provided robust protection against both viruses, although levels of virus in BAL and nasal swabs were higher after B.1.351 challenge than after WA1/2020 challenge.

To the best of our knowledge, this is the first report of a SARS-CoV-2 vaccine evaluated for efficacy against a SARS-CoV-2 variant of concern in macaques. Several SARS-CoV-2 vaccines have previously been reported to protect against homologous WA1/2020 challenges, but have not yet been reported against B.1.351 challenges. Our study does not define mechanistic correlates of protection against SARS-CoV-2 variants, but it has previously been reported that IgG was sufficient for protection against homologous SARS-CoV-2 challenge in macaques and that CD8 T cell responses also contributed to protection if antibody titres were subprotective^22^.

In conclusion, Ad26.COV2.S induced cross-reactive humoral and cellular immune responses and provided robust protection against the heterologous SARS-CoV-2 variant B.1.351 in rhesus macaques. Future studies will determine whether Ad26.COV2.S, as well as other vaccines, protect against other SARS-CoV-2 variants of concern.

## Methods

No statistical methods were used to predetermine sample size. Macaques were randomized into groups. All immunological, virological and histopathological studies were performed blinded.

### Macaques and study design

Twenty-four outbred Indian-origin adult male and female rhesus macaques (*Macaca mulatta*) (3–11 years old) were randomly allocated to groups. All macaques were housed at Bioqual. Macaques received a single immunization of 5 × 10^10^ viral particles of Ad26.COV2.S (*n* = 12) or sham (*n* = 12) by the intramuscular route without adjuvant at week 0. At week 6, all macaques were challenged with 5 × 10^5^ TCID_50_ SARS-CoV-2 from strains USA-WA1/2020 (BEI Resources; NR-5228) (which was grown in VeroE6 cells and deep sequenced as previously described^16^) or B.1.351 (BEI Resources; NR-54974). The B.1.351 stock was grown in Calu-3 cells and was deep-sequenced, which confirmed the expected sequence identity with no mutations in the S greater than 2.5% frequency and no mutations elsewhere in the virus at greater than 13% frequency. Virus was administered as 1 ml by the intranasal route (0.5 ml in each nare) and 1 ml by the intratracheal route. All immunological, virological and histopathological studies were performed blinded. Animal studies were conducted in compliance with all relevant local, state and federal regulations and were approved by the Bioqual Institutional Animal Care and Use Committee.

### Pseudovirus-based virus neutralization assay

The SARS-CoV-2 pseudoviruses expressing a luciferase reporter gene were generated essentially as previously described^12,16–18^. In brief, the packaging plasmid psPAX2 (AIDS Resource and Reagent Program), luciferase reporter plasmid pLenti-CMV Puro-Luc (Addgene) and S expressing pcDNA3.1-SARS CoV-2 SΔCT of variants were co-transfected into HEK293T cells by lipofectamine 2000 (ThermoFisher). Pseudoviruses of SARS-CoV-2 variants were generated by using WA1/2020 strain (Wuhan/WIV04/2019, GISAID accession identifier EPI_ISL_402124), D614G mutation, B.1.1.7 variant (GISAID accession identifier EPI_ISL_601443) or B.1.351 variant (GISAID accession identifier EPI_ISL_712096). The supernatants containing the pseudotype viruses were collected 48 h after transfection, and were purified by centrifugation and filtration with a 0.45-μm filter. To determine the neutralization activity of the plasma or serum samples from participants, HEK293T cells expressing human ACE2 (HEK293-hACE2 cells) were seeded in 96-well tissue culture plates at a density of 1.75 × 10^4^ cells per well overnight. Threefold serial dilutions of heat-inactivated serum or plasma samples were prepared and mixed with 50 μl of pseudovirus. The mixture was incubated at 37 °C for 1 h before adding to HEK293T-hACE2 cells. Forty-eight hours after infection, cells were lysed in Steady-Glo Luciferase Assay (Promega) according to the manufacturer’s instructions. SARS-CoV-2 neutralization titres were defined as the sample dilution at which a 50% reduction in relative light units (RLU) was observed relative to the average of the virus control wells.

### Live virus neutralization assay

Full-length SARS-CoV-2 WA1/2020, B.1.351 and B.1.1.7, viruses were designed to express nanoluciferase (nLuc) and were recovered via reverse genetics^19^. One day before the assay, Vero E6 USAMRID cells were plated at 20,000 cells per well in clear-bottom black-walled plates. Cells were inspected to ensure confluency on the day of assay. Serum samples were tested at a starting dilution of 1:20 and were serially diluted threefold up to nine dilution spots. Serially diluted serum samples were mixed in equal volume with diluted virus. Antibody–virus and virus-only mixtures were then incubated at 37 °C with 5% CO_2_ for 1 h. After incubation, serially diluted sera and virus-only controls were added in duplicate to the cells at 75 plaque-forming units at 37 °C with 5% CO_2_. Twenty-four hours later, the cells were lysed, and luciferase activity was measured via Nano-Glo Luciferase Assay System (Promega) according to the manufacturer specifications. Luminescence was measured by a Spectramax M3 plate reader (Molecular Devices). Virus neutralization titres were defined as the sample dilution at which a 50% reduction in RLU was observed relative to the average of the virus control wells.

### ELISA

WA1/2020, B.1.1.7 and B.1.351 RBD-specific binding antibodies were assessed by ELISA essentially as previously described^12,16,17^. In brief, 96-well plates were coated with 0.5 μg ml^−1^ RBD protein in 1× DPBS and incubated at 4 °C overnight. After incubation, plates were washed once with wash buffer (0.05% Tween-20 in 1× DPBS) and blocked with 350 μl casein block per well for 2–3 h at room temperature. After incubation, block solution was discarded and plates were blotted dry. Serial dilutions of heat-inactivated serum diluted in casein block were added to wells and plates were incubated for 1 h at room temperature, before three further washes and a 1-h incubation with a 1 μg ml^−1^ dilution of anti-macaque IgG HRP (Nonhuman Primate Reagent Resource) at room temperature in the dark. Plates were then washed three times, and 100 μl of SeraCare KPL TMB SureBlue Start solution was added to each well; plate development was halted by the addition of 100 μl SeraCare KPL TMB Stop solution per well. The absorbance at 450 nm was recorded using a VersaMax microplate reader. For each sample, ELISA endpoint titre was calculated in GraphPad Prism software, using a four-parameter logistic curve fit to calculate the reciprocal serum dilution that yields an absorbance value of 0.2 at 450 nm. log_10_-transformed endpoint titres are reported.

### ECLA

ECLA plates (MesoScale Discovery SARS-CoV-2 IgG Cat No: N05CA-1; panel 7) were designed and produced with up to nine antigen spots in each well, and assays were performed essentially as previously described^20^. The antigens included were WA1/2020, B.1.1.7, P.1, and B.1.351 S and RBD. The plates were blocked with 50 μl of blocker A (1% BSA in MilliQ water) solution for at least 30 m at room temperature shaking at 700 rpm with a digital microplate shaker. During blocking, the serum was diluted 1:5,000 in diluent 100. The plates were then washed 3 times with 150 μl of the MSD kit wash buffer, blotted dry, and 50 μl of the diluted samples were added in duplicate to the plates and set to shake at 700 rpm at room temperature for at least 2 h. The plates were again washed 3 times and 50 μl of SULFO-tagged anti-human IgG detection antibody (MesoScale Discovery) diluted to 1× in diluent 100 was added to each well and incubated shaking at 700 rpm at room temperature for at least 1 h. Plates were then washed 3 times and 150 μl of MSD GOLD read buffer B was added to each well and the plates were read immediately after on a MESO QuickPlex SQ 120 machine. MSD titres for each sample are reported as RLUs, which were calculated as sample RLU minus blank RLU for each spot for each sample. The limit of detection was defined as 1,000 RLU for each assay.

### Fc functional antibody assays

Fc functional profiling included the assessment of antibody-dependent monocyte phagocytosis and antibody-dependent complement deposition^21^. In brief, fluorescent beads (LifeTechnologies) were coupled via carboxy-coupling, and plasma were added, allowing immune complex formation, excess antibodies were washed away, followed by the addition of THP1 monocytes, primary neutrophils or guinea pig complement, individually, respectively. The level of phagocytosis and complement deposition was assessed by flow cytometry.

### IFNγ ELISPOT assay

Pooled peptide ELISPOT assays were performed essentially as previously described^12,16,17^. Peptide pools consisted of 15 amino acid peptides overlapping by 11 amino acids spanning the SARS-CoV-2 S from the WA1/2020 strain or variant strains. ELISPOT plates were coated with mouse anti-human IFNγ monoclonal antibody from BD Pharmigen at 5 μg per well and incubated overnight at 4 °C. Plates were washed with DPBS wash buffer (DPBS with 0.25% Tween-20), and blocked with R10 medium (RPMI with 10% heat-inactivated FBS with 1% of 100× penicillin–streptomycin) for 1–4 h at 37 °C. SARS-CoV-2 peptides (21st Century Biochemicals) were prepared and plated at a concentration of 1 μg per well, and 200,000 cells per well were added to the plate. The peptides and cells were incubated for 18–24 h at 37 °C. All steps after this incubation were performed at room temperature. The plates were washed with ELISPOT wash buffer (11% 10× DPBS and 0.3% Tween-20 in 1 l MilliQ water) and incubated for 2 h with rabbit polyclonal anti-human IFNγ biotin from U-Cytech (1 μg ml^−1^). The plates were washed a second time and incubated for 2 h with streptavidin–alkaline phosphatase from Southern Biotech (2 μg ml^−1^). The final wash was followed by the addition of nitor-blue tetrazolium chloride or 5-bromo-4-chloro 3′ indolyphosphate *p*-toludine salt (NBT/BCIP chromagen) substrate solution for 7 min. The chromagen was discarded and the plates were washed with water and dried in a dim place for 24 h. Plates were scanned and counted on a Cellular Technologies Limited Immunospot Analyzer.

### Intracellular cytokine staining assay

Multi-parameter pooled-peptide intracellular cytokine staining assays were performed essentially as previously described^12,16,17^. Peptide pools consisted of 15 amino acid peptides overlapping by 11 amino acids spanning the SARS-CoV-2 S from the WA1/2020 strain or variant strains. Then, 10^6^ PBMCs per well were resuspended in 100 μl of R10 medium supplemented with CD49d monoclonal antibody (1 μg ml^−1^). Each sample was assessed with mock (100 μl of R10 plus 0.5% DMSO; background control), peptide pools (2 μg ml^−1^), or 10 pg ml^−1^ phorbol myristate acetate and 1 μg ml^−1^ ionomycin (Sigma-Aldrich) (100 μl; positive control) and incubated at 37 °C for 1 h. After incubation, 0.25 μl of GolgiStop and 0.25 μl of GolgiPlug in 50 μl of R10 was added to each well and incubated at 37 °C for 8 h and then held at 4 °C overnight. The next day, the cells were washed twice with DPBS, stained with near-IR live/dead dye for 10 min and then stained with predetermined titres of monoclonal antibodies against CD279 (clone EH12.1, BB700), CD38 (clone OKT10, PE), CD28 (clone 28.2, PE CY5), CD4 (clone L200, BV510), CD95 (clone DX2, BUV737) and CD8 (clone SK1, BUV805), for 30 min. Cells were then washed twice with 2% FBS in DPBS buffer and incubated for 15 min with 200 μl of BD CytoFix/CytoPerm Fixation/Permeabilization solution. Cells were washed twice with 1× Perm Wash buffer (BD Perm/Wash Buffer 10× in the CytoFix/CytoPerm Fixation/Permeabilization kit diluted with MilliQ water and passed through a 0.22-μm filter) and stained with intracellularly with monoclonal antibodies against Ki67 (clone B56, FITC), CD69 (clone TP1.55.3, ECD), IL-10 (clone JES3-9D7, PE CY7), IL-13 (clone JES10-5A2, BV421), TNF (clone Mab11, BV650), IL-4 (clone MP4-25D2, BV711), IFNγ (clone B27; BUV395), CD45 (clone D058-1283, BUV615), IL-2 (clone MQ1-17H12, APC) and CD3 (clone SP34.2, Alexa 700), for 30 min. Cells were washed twice with 1× Perm Wash buffer and fixed with 250 μl of freshly prepared 1.5% formaldehyde. Fixed cells were transferred to a 96-well round-bottom plate and analysed by BD FACSymphony system. Central memory T cells were defined as CD28^+^CD95^+^ T cells. Data were analysed with FlowJo v.9.9.

### sgRNA assay

SARS-CoV-2 *E* gene sgRNA was assessed by RT–PCR using primers and probes as previously described^23,24^. A standard was generated by first synthesizing a gene fragment of the subgenomic *E* gene^23^. The gene fragment was subsequently cloned into a pcDNA3.1+ expression plasmid using restriction site cloning (Integrated DNA Techonologies). The insert was in vitro-transcribed to RNA using the AmpliCap-Max T7 High Yield Message Maker Kit (CellScript). log dilutions of the standard were prepared for RT–PCR assays ranging from 1 × 10^10^ copies to 1 × 10^−1^ copies. Viral loads were quantified from BAL fluid and nasal swabs. RNA extraction was performed on a QIAcube HT using the IndiSpin QIAcube HT Pathogen Kit according to manufacturer’s specifications (Qiagen). The standard dilutions and extracted RNA samples were reverse-transcribed using SuperScript VILO Master Mix (Invitrogen) following the cycling conditions described by the manufacturer, 25 °C for 10 min, 42 °C for 1 h, then 85 °C for 5 min. A Taqman custom gene expression assay (Thermo Fisher Scientific) was designed using the sequences targeting the *E* gene sgRNA^23^. The sequences for the custom assay were as follows, forward primer, sgLeadCoV2.Fwd: CGATCTCTTGTAGATCTGTTCTC, E_Sarbeco_R: ATATTGCAGCAGTACGCACACA, E_Sarbeco_P1 (probe): VIC-ACACTAGCCATCCTTACTGCGCTTCG-MGB. These primers and probes were equally reactive for both variants. Reactions were carried out in duplicate for samples and standards on the QuantStudio 6 and 7 Flex Real-Time PCR Systems (Applied Biosystems) with the thermal cycling conditions, initial denaturation at 95 °C for 20 s, then 45 cycles of 95 °C for 1 s and 60 °C for 20 s. Standard curves were used to calculate sgRNA copies per ml or per swab; the quantitative assay sensitivity was 50 copies per ml or per swab.

### TCID_50_ assay

Vero TMPRSS2 cells (obtained from A. Creanga) were plated at 25,000 cells per well in DMEM with 10% FBS and gentamicin, and the cultures were incubated at 37 °C, 5.0% CO_2_. Medium was aspirated and replaced with 180 μl of DMEM with 2% FBS and gentamicin. Serial dilution of samples as well as positive (virus stock of known infectious titre) and negative (medium only) controls were included in each assay. The plates are incubated at 37 °C, 5.0% CO_2_ for 4 days. Cell monolayers were visually inspected for cytopathic effect. The TCID_50_ was calculated using the Read–Muench formula.

### Histopathology

Lungs on day 10 after SARS-CoV-2 challenge were evaluated by histopathology. At the time of fixation, lungs were suffused with 10% formalin to expand the alveoli. All tissues were fixed in 10% formalin and blocks sectioned at 5 μm. Slides were incubated for 30–60 min at 65 °C then deparaffinized in xylene and rehydrated through a series of graded ethanol to distilled water. Sections were stained with haematoxylin and eosin. Blinded evaluation and scoring was performed by a board-certified veterinary pathologist (A.J.M.).

### Statistical analyses

Comparisons of virological, immunological and histopathological data were performed using GraphPad Prism 8.4.2 (GraphPad Software). Comparison of data between groups was performed using two-sided Wilcoxon rank-sum tests. Correlation analyses were performed using two-sided Spearman rank-correlation tests. *P* values of less than 0.05 were considered significant.

### Reporting summary

Further information on research design is available in the Nature Research Reporting Summary linked to this paper.

## Online content

Any methods, additional references, Nature Research reporting summaries, source data, extended data, supplementary information, acknowledgements, peer review information; details of author contributions and competing interests; and statements of data and code availability are available at 10.1038/s41586-021-03732-8.

## Supplementary information

Supplementary Figure 1 Sample Raw Flow Cytometry Data.

Reporting Summary

## Data Availability

All relevant data are available in the Article and its Supplementary Information. Any additional data are available from the corresponding author upon reasonable request. Source data are provided with this paper.

## Source data

Source Data Fig. 1
Source Data Fig. 2
Source Data Fig. 3
Source Data Fig. 4
Source Data Fig. 5
