# Supplementary material for: Protective efficacy of Ad26.COV2.S against SARS-CoV-2 B.1.351 in macaques
Source: Nature. 2021 Jun 23;596(7872):423–7. doi: 10.1038/s41586-021-03732-8 (PMC8373608; doi:10.1038/s41586-021-03732-8)
Supplement: Supplementary file 1 — Sample Raw Flow Cytometry Data. [file 41586_2021_3732_MOESM1_ESM.pdf]

nature portfolio
https://doi.org/10.1038/s41586-021-03732-8

Supplementary information

Protective efficacy of Ad26.COV2.S against SARS-CoV-2 B.1.351 in macaques

In the format provided by the authors and unedited

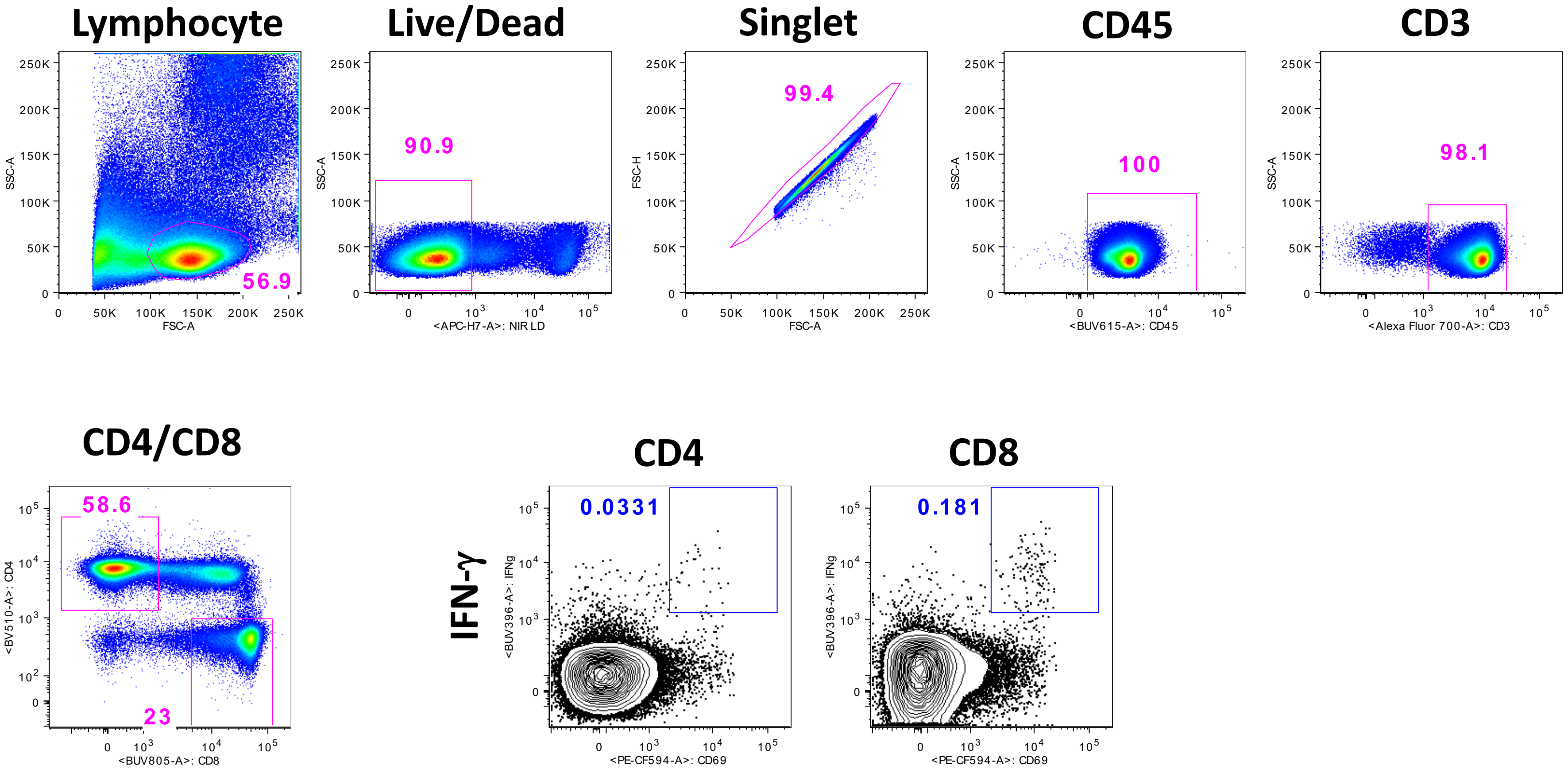

Supplemental Figure 1. Sample Raw Flow Cytometry Data.
