# Supplementary material for: Protective efficacy of Ad26.COV2.S against SARS-CoV-2 B.1.351 in macaques
Source: Nature. 2021 Jun 23;596(7872):423–7. doi: 10.1038/s41586-021-03732-8 (PMC8373608; doi:10.1038/s41586-021-03732-8)
Supplement: Supplementary file 2 — Reporting Summary [file 41586_2021_3732_MOESM2_ESM.pdf]

nature research

Corresponding author(s): Dan H. Barouch

Last updated by author(s): Jun 12, 2021

Reporting Summary

Nature Research wishes to improve the reproducibility of the work that we publish. This form provides structure for consistency and transparency in reporting. For further information on Nature Research policies, see our Editorial Policies and the Editorial Policy Checklist.

Statistics

For all statistical analyses, confirm that the following items are present in the figure legend, table legend, main text, or Methods section.

n/a	Confirmed	
☐	☒	The exact sample size (n) for each experimental group/condition, given as a discrete number and unit of measurement
☐	☒	A statement on whether measurements were taken from distinct samples or whether the same sample was measured repeatedly
☐	☒	The statistical test(s) used AND whether they are one- or two-sided<br>*Only common tests should be described solely by name; describe more complex techniques in the Methods section.*
☐	☒	A description of all covariates tested
☐	☒	A description of any assumptions or corrections, such as tests of normality and adjustment for multiple comparisons
☐	☒	A full description of the statistical parameters including central tendency (e.g. means) or other basic estimates (e.g. regression coefficient) AND variation (e.g. standard deviation) or associated estimates of uncertainty (e.g. confidence intervals)
☐	☒	For null hypothesis testing, the test statistic (e.g. F, t, r) with confidence intervals, effect sizes, degrees of freedom and P value noted<br>*Give P values as exact values whenever suitable.*
☒	☐	For Bayesian analysis, information on the choice of priors and Markov chain Monte Carlo settings
☒	☐	For hierarchical and complex designs, identification of the appropriate level for tests and full reporting of outcomes
☒	☐	Estimates of effect sizes (e.g. Cohen's d, Pearson's r), indicating how they were calculated

Our web collection on statistics for biologists contains articles on many of the points above.

Software and code

Policy information about availability of computer code

Data collection	QuantStudio 6 was used to collect sgRNA data.
Data analysis	Analysis of virologic and immunologic data was performed using GraphPad Prism 8.4.2 (GraphPad Software). Flow cytometry data was analyzed with FlowJo v9.9.

For manuscripts utilizing custom algorithms or software that are central to the research but not yet described in published literature, software must be made available to editors and reviewers. We strongly encourage code deposition in a community repository (e.g. GitHub). See the Nature Research guidelines for submitting code & software for further information.

Data

Policy information about availability of data

All manuscripts must include a data availability statement. This statement should provide the following information, where applicable:

- Accession codes, unique identifiers, or web links for publicly available datasets
- A list of figures that have associated raw data
- A description of any restrictions on data availability

All data are available in the manuscript or the supplementary material.

Field-specific reporting

Please select the one below that is the best fit for your research. If you are not sure, read the appropriate sections before making your selection.

☒ Life sciences ☐ Behavioural & social sciences ☐ Ecological, evolutionary & environmental sciences

For a reference copy of the document with all sections, see nature.com/documents/nr-reporting-summary-flat.pdf

Life sciences study design

All studies must disclose on these points even when the disclosure is negative.

Sample size	Sample size includes N=24 vaccinated animals (N=6 animals/group; Mercado et al Nature 2020). Based on our experience with SARS-CoV-2 in rhesus macaques, this sample size can differentiate large differences in protective efficacy compared with the sham controls.
Data exclusions	No data were excluded.
Replication	Virologic and immunologic measures were performed in duplicate. Technical replicates were minimally different. All attempts at replication were successful.
Randomization	Animals were balanced for age and gender and otherwise randomly allocated to groups.
Blinding	All immunologic and virologic assays were performed blinded.

Reporting for specific materials, systems and methods

We require information from authors about some types of materials, experimental systems and methods used in many studies. Here, indicate whether each material, system or method listed is relevant to your study. If you are not sure if a list item applies to your research, read the appropriate section before selecting a response.

Materials & experimental systems

n/a	Involved in the study	
☐	☒	Antibodies
☐	☒	Eukaryotic cell lines
☒	☐	Palaeontology and archaeology
☐	☒	Animals and other organisms
☒	☐	Human research participants
☒	☐	Clinical data
☒	☐	Dual use research of concern

Methods

n/a	Involved in the study	
☒	☐	ChIP-seq
☐	☒	Flow cytometry
☒	☐	MRI-based neuroimaging

Antibodies

Antibodies used	For ELISA and ELISPOT assays anti-macaque IgG HRP (NIH NHP Reagent Program), rabbit polyclonal anti-human IFN-γ (U-Cytech); for ICS assays mAbs against CD279 (clone EH12.1, BB700), CD38 (clone OKT10, PE), CD28 (clone 28.2, PE CY5), CD4 (clone L200, BV510), CD45 (clone D058-1283, BUV615), CD95 (clone DX2, BUV737), CD8 (clone SK1, BUV805), Ki67 (clone B56, FITC), CD69 (clone TP1.55.3, ECD), IL10 (clone JES3-9D7, PE CY7), IL13 (clone JES10-5A2, BV421), TNF-α (clone Mab11, BV650), IL4 (clone MP4-25D2, BV711), IFN-γ (clone B27; BUV395), IL2 (clone MQ1-17H12, APC), CD3 (clone SP34.2, Alexa 700) (BD); for 800CW-conjugated goat-anti-human secondary antibody (Li-COR); anti-rhesus IgG1, IgG2, IgG3, IgA, IgM (NIH NHP Reagent Program); tertiary goat anti-mouse IgG-PE antibody (Southern Biotech), anti-CD107a (PE-Cy7, BD), anti-CD56 (PE-Cy7, BD), anti-MIP-1β (PE, BD), mouse anti-human IFN-γ monoclonal antibody (BD), Streptavidin-alkaline phosphatase antibody (Southern Biotech), CD49d (BD), sulfo-tagged anti-human IgG (MesoScale Discovery).
Validation	all mAbs used according to manufacturer's instructions and were titrated prior to use

Eukaryotic cell lines

Policy information about cell lines

Cell line source(s)	None
Authentication	Commerically purchased (ATCC) and evaluated in control experiments prior to use
Mycoplasma contamination	Negative for mycoplasma

Commonly misidentified lines (See ICLAC register)	None were utilized

Animals and other organisms

Policy information about studies involving animals; ARRIVE guidelines recommended for reporting animal research

Laboratory animals	24 outbred Indian-origin adult male and female rhesus macaques (Macaca mulatta), 3-11 years old
Wild animals	None
Field-collected samples	None
Ethics oversight	Bioqual IACUC

Note that full information on the approval of the study protocol must also be provided in the manuscript.

Flow Cytometry

Plots

Confirm that:

☒ The axis labels state the marker and fluorochrome used (e.g. CD4-FITC).

☒ The axis scales are clearly visible. Include numbers along axes only for bottom left plot of group (a 'group' is an analysis of identical markers).

☒ All plots are contour plots with outliers or pseudocolor plots.

☒ A numerical value for number of cells or percentage (with statistics) is provided.

Methodology

Sample preparation	Isolated PBMC
Instrument	BD FACSymphony
Software	FlowJo v9.9
Cell population abundance	No sorting was performed
Gating strategy	See gating strategy in Supplementary Figure 1

☒ Tick this box to confirm that a figure exemplifying the gating strategy is provided in the Supplementary Information.
